# Optimized Design of a Pump Laser System for a Spin Exchange Relaxation Free Inertial Measurement Device

**DOI:** 10.3390/s21092982

**Published:** 2021-04-23

**Authors:** Jian Hao, Hong-Liang Ke, Zhai-Yue Yang, Bang-Cheng Han

**Affiliations:** 1 Zhejiang Lab, Research Center for Quantum Sensing, Hangzhou 311100, China; haojian@zhejianglab.com (J.H.); zhaiyueyang@zhejianglab.com (Z.-Y.Y.); hanbangcheng@buaa.edu.cn (B.-C.H.); 2 Hangzhou Innovation Institute, Beihang University, Hangzhou 310000, China; 3 School of Instrumentation and Optoelectronic Engineering, Beihang University, Beijing 100191, China; 4 Beijing Advanced Innovation Center for Big Data-Based Precision Medicine, Beihang University, Beijing 100083, China

**Keywords:** square wave modulation, stability control of power, beam shaping design, freeform surface lens

## Abstract

In order to improve the precision and beam quality of a pump laser for a spin exchange relaxation free inertial measurement device, we applied one scheme to achieve the square wave modulation and power stability control of the pump laser and another one to obtain the uniform intensity distribution of the laser beam, in which the acousto-optic modulator (AOM) and proportion integration differentiation (PID) controller were used to achieve the former, and the freeform surface lens was designed and optimized to achieve the latter based on the TracePro software. In experiments, the first-order diffraction light beam coming through the AOM had a spot size of about 1.1 × 0.7 mm^2^, and a spherical vapor cell with a radius of 7 mm was placed behind the freeform surface lens. Results show that the uniformity of the reshaped intensity distribution is higher than 90% within the target area with a radius of 7 mm both in the simulation and the experiment, which ensure that the uniform laser beam covers the area of cell. On the other hand, the power stability of the pump laser is controlled to be less than 0.05%. Compared with traditional methods, the complicated calculation process in optical design is better solved, and a higher uniformity with slight energy loss is achieved.

## 1. Introduction

An alkali-metal-noble-gas device has found applications in the measurement of inertial rotation [1,2,3], Lorentz and CPT violation [4,5], anomalous spin forces [6,7], etc. In the K-Rb-^21^Ne inertial measurement device, Rb atoms are operated in the spin-exchange-relaxation-free (SERF) regime [8], where the effect of spin-exchange collisions is slight [9], so the inertial measurement device was developed and experimentally proved. The main principle is to polarize K atoms by a pump laser of 770 nm, and then Rb atoms are polarized by spin-exchange collisions of K atoms. When the inertial measurement device rotates at a certain angular velocity, the spin state of Rb atoms changes accordingly. The spin state of Rb atoms is detected by a probe laser of 795 nm, and then the rotation angle is extracted. Therefore, the pump method and power stability of the laser [10] and the spot size and distribution of the light intensity of the pump laser [11] can both have an effect on the polarization efficiency of K and Rb atoms, and thus on the detection accuracy.

In terms of the study of the pump method and power stability for the pump laser, compared with the pump method of non-modulation [12,13] and sinusoidal modulation [14,15], the pump method of square wave modulation [16,17,18] is more suitable for mobile platforms with the characteristics of less influence from the external environment, high data repeatability and high response frequency. The power stabilization method of the pump laser includes the electric control method [19], the electro-optic modulator method [20], liquid crystal variable retarder method [21], etc., while the acousto-optic modulator method not only gives a high extinction ratio, but can also modulate the first-order diffraction light with the opening and closing signal [16]. As a result, the AOM can be used to achieve both power stabilization and square wave modulation.

In terms of the study of spot size and intensity distribution of the pump laser, a beam expander or lens group is always used to expand the laser spot, as to make sure that the spot size is larger than the size of the gas vapor [22,23,24,25], in which only the relatively uniform light in the center of the whole spot is applied [11]. However, most of the light energy is wasted in this method, and the ideal point laser source or Gaussian beam is always taken to be considered in the optical arrangement, not the actual sources such as the asymmetric laser source or other non-ideal light sources. Most importantly, the incident angle of the laser passing through the vapor cell is not considered, which causes the refraction or reflection of light, and thereby results in a light shift or non-uniform intensity distribution. Compared with the other beam shaping methods [26,27,28,29,30], the method with a freeform surface lens [31,32] with a high degree of freedom and less energy loss is better for achieving the uniform distribution of intensity of non-ideal laser sources. At shorter distances, a better uniform intensity distribution can be achieved by using only one freeform surface lens [33], especially for a big spherical vapor cell.

In this paper, we applied one scheme to achieve the square wave modulation and the power stability of a pump laser, and another one to get the uniform intensity distribution of a laser beam, in which the acousto-optic modulator (AOM) and proportion integration differentiation (PID) controller are used to achieve the former, and the freeform surface lens is designed and optimized to achieve the latter based on the TracePro software.

## 2. Experiments

**Figure 1:**
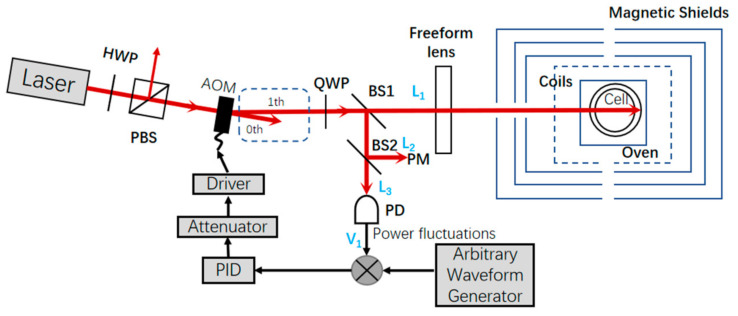
Arrangement of the system.

The arrangement of the system is shown in Figure 1. The total power of the laser was adjusted by a half-wave plate (HWP) and polarization beam splitting (PBS), and the mode of square wave modulation of the laser was realized by the first-order diffraction light of the AOM controlled by a switch driving signal. The center frequency of the AOM was 80 MHz, and the applied voltage amplitude was 0~1 V. A quarter-wave plate (QWP) was used to convert linearly polarized light to circularly polarized light, which in turn polarized alkali metal atoms in the gas cell. By passing through the beam splitting (BS_1_ and BS_2_), the total light was divided into L_1_, L_2_ and L_3_. L_1_, used for reshaping the beam by the designed freeform lens and then pumping the atoms. L_3_ was measured by a photoelectric detector (PD), and its value was required to be less than 10mW after beam splitting.

The power stabilization system was designed as follows. Firstly, a square wave voltage signal was applied to the driver of the AOM by an arbitrary waveform generator, and thereby the pulsed light was produced. Secondly, in the case of high voltage, the voltage V detected by PD and the power L_1_ detected by the power meter were extracted. Thirdly, in the case of an open-loop system, the linear relationship between V and L_1_ was obtained. Fourthly, the reference signal V_0_ was calculated with the obtained linear relationship, with the given power signal L_0_ required by the system. Fifthly, the error signal was calculated based on the reference signal V_0_ and the actual signal V_1_. Sixthly, a proportion integration differentiation (PID) circuit and an attenuator were used to compensate for the error signal, in which the PID controller, namely a noise eater (TEM Messtechnik, NoiseEater 3V2) was applied, and the proportional, derivative, and integral gains were adjusted to reach a proper working point by trimming the knobs on the noise eater. The working point of the PID was thereby achieved by injecting a voltage signal of an arbitrary waveform generator. Finally, the compensated signal was processed into a square wave signal by the driver. As a result, the diffraction efficiency of the AOM was then changed and the stability of power was well controlled. The other light L_2_ was used for a long-term power stability measurement by power meter (PM). The power stability can be defined as follows:

(1) $$S=\pm \frac{P_{\max}-P_{\min}}{\bar{P}}\times 100\%$$

where, *P*_max_ and *P*_min_ are, respectively, the maximum and minimum value of output power during testing, and $\bar{P}$ is the average value. The uniformity of the spot can be calculated as follows [31]:

(2) $$E_{\mathrm{uniformity}}=\left(1-\sqrt{\frac{\sum_{i=1}^{M}{\left(E_{i}-\bar{E}\right)}^{2}}{M{\bar{E}}^{2}}}\right)\times 100\%$$

where *M* is the number of sampling points, *E (W/m*^2^*)* = *P (W) /A (m*^2^*)*, *P* is the laser power, *A* is the target area, *E_i_* is the corresponding value at each point, and $\bar{E}$ is the averaged value.

## 3. Pump Method and Power Stability

Before reshaping the intensity distribution of the spot, the laser power was controlled to be stabilized by the method above. A 770 nm laser (UniQuanta Company) was selected as the pump laser source. The spot size was about 0.55 × 1.35 mm^2^, and the maximum power was 2 W. The AOM (Gooch & Housego Company, 3080-125), the beam quality analyzer (Thorlabs Company, BC106N-VIS/M), the photodetector (Thorlabs Company, PDA100A2), the power meter (Thorlabs Company, PM100D), and oscilloscope (Tektronix Company, TDS2024C) were used in the experiments.

In the experiment, the laser power was set to 600 mW. The beam splitting ratio (transmittance: reflectivity) of BS_1_ and BS_2_ was selected to be 98:20 and 30:70, respectively. As a result, L_1_, L_2_ and L_3_ were about 540 mW, 7.6 mW, and 3.3 mW, respectively. For a better presentation of the optical power data, the frequency and duty ratio of square wave modulation were set to 10 MHz and 95%, respectively. However, a good result was usually obtained with a frequency lower than MHz in practice. In the experiments of the inertial measurement device, the setting of the two parameters were determined by the number of atoms of a gas cell and the relaxation time.

**Figure 2:**
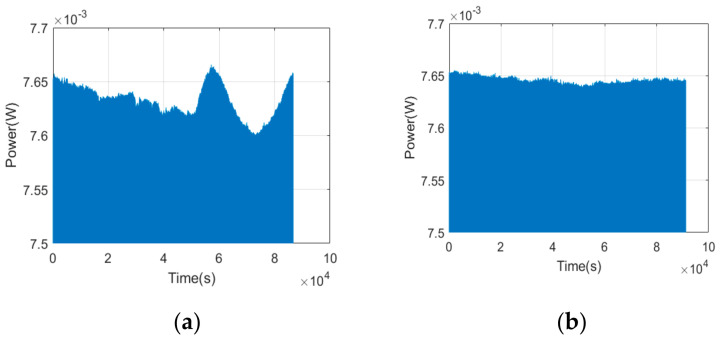
Variation of L_2_ over time in the case without PID (**a**), and in the case with PID (**b**).

**Figure 3:**
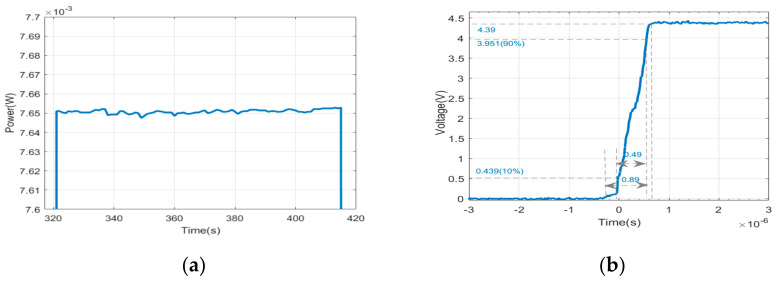
Variation of L2 during a single pulse of the square wave modulation measured by a power meter (**a**), and by an oscilloscope (**b**).

The variation of L_2_ during a 24 h long-time test in the case without PID and with PID is shown in Figure 2a,b, respectively, and the time resolution was 1s. The calculated power stability was 0.91% and 0.13%, respectively, according to Equation (1). Figure 3a shows the variation of L_2_ during a single pulse of the square wave modulation. Apparently, the rising time and response time of the single pulse can not be obtained owing to the limited sampling frequency of 1/s of power meter, and we obtained the results by using an oscilloscope with a sampling frequency of 2 GS/s, as shown in Figure 3b. The rise time and response time were measured to be 0.89 × 10^−6^ s and 0.49 × 10^−6^ s, respectively.

## 4. Intensity Distribution of the Spot

The optimization design of the pump laser includes the simulation of a substitute light source, calculation of aperture for a freeform surface lens and optimization design of a freeform surface lens.

### 4.1. Simulation of Substitute Light Source

**Figure 4:**
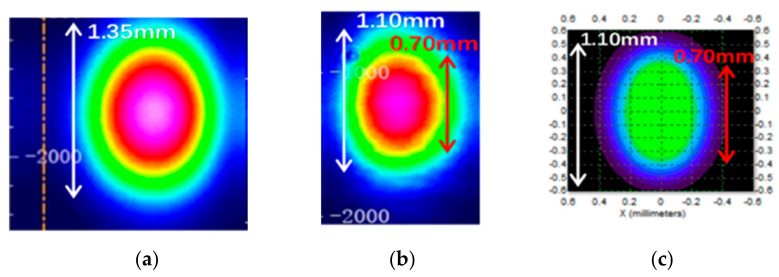
Shape of the spot obtained before diffraction (**a**), after diffraction (**b**) in the experiment. Shape of the simulated first-order spot (**c**) in simulation.

**Figure 5:**
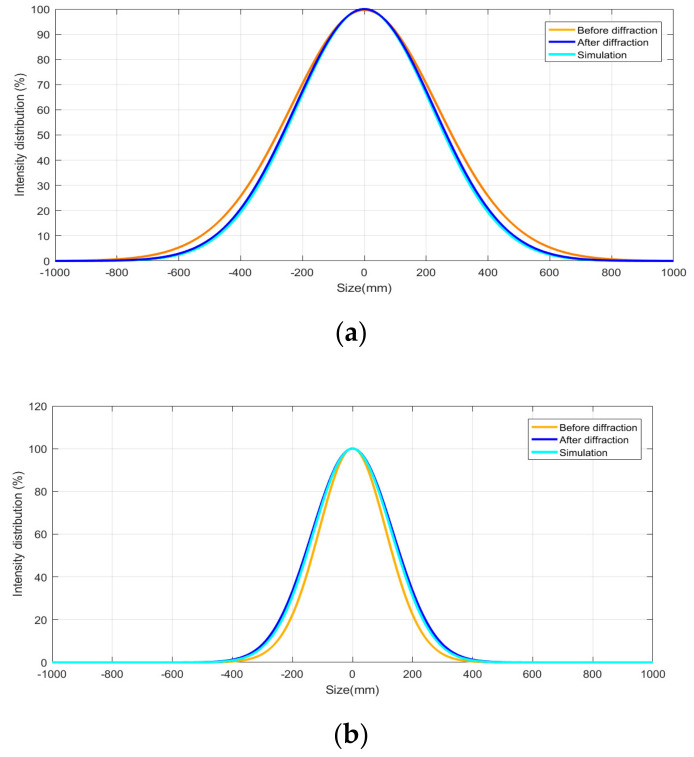
Intensity distribution of the first-order diffraction laser along the longitudinal direction (**a**) and along the transverse direction (**b**).

After passing through the AOM, the two laser spots were formed, namely the first-order diffraction light and the zero-order diffraction light. The energy distribution of these two spots was controlled by the diffraction efficiency of the AOM, resulting in the differences between the input laser and the first-order diffraction laser in the energy and size. The spot shape of the input laser and the first-order light were measured by a beam quality analyzer and shown in Figure 4a,b, and the longitudinal lengths of the spot were 1.35 mm and 1.10 mm, respectively. The spot size of the first-order diffraction laser was about 0.70 × 1.10 mm^2^. The ellipticity of the first-order diffraction light spot was 88.9% and the diffraction efficiency was higher than 80%. Centering on the highest intensity value of the two spots, the intensity value along the longitudinal and transverse directions was normalized and fitted by the Gaussian distribution in the software of the beam quality analyzer, as shown in Figure 5a,b. The brown and blue curves represent the intensity distribution of the laser spot before diffraction, and that of the first-order laser spot after diffraction, respectively. The detection surface was placed 80 mm away from the AOM.

Apparently, the longitudinal size of the spot changes from 1.35 mm to 1.10 mm after diffraction. Therefore, a method of light source substitution was proposed, in which a new light source is established to replace the square wave modulation part that included the laser, HWP, PBS, and the AOM. The AOM was placed behind the beam waist of the laser. Then the attributes of the light source were established in TracePro software by using the function of surface light editor. The wavelength and angle distributions of the laser source were both selected as Gaussian distribution, and the center wavelength was set at 770 nm. The simulated spot is shown in Figure 4c. For the light source points with different intensity values, separate values can be assigned. It can be seen that the shape of the spot is basically the same. For the mismatch between the simulated values and tested values, a separate value can be assigned in TracePro. Centering on the highest intensity value of the simulated spot, the intensity value along longitudinal and transverse directions is normalized and fitted by using Gaussian distribution in the same way, as shown in the light blue curve of Figure 5a,b, in which the detection surface was placed just behind the simulated light source.

**Figure 6:**
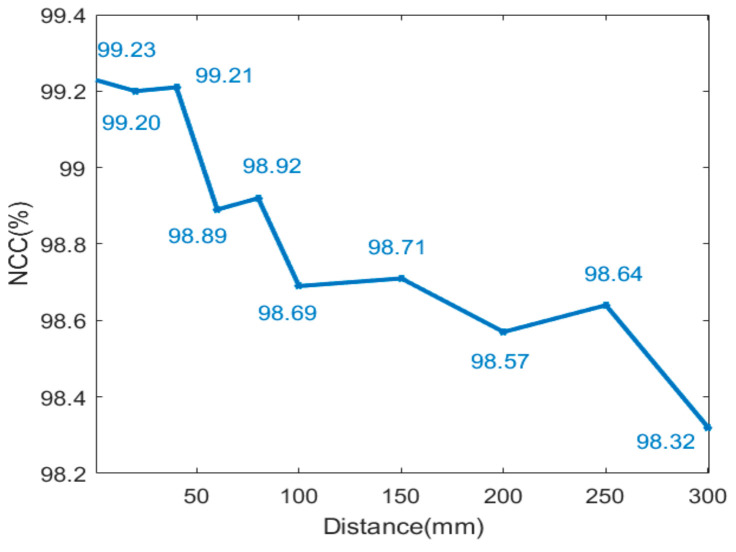
The NCC of the intensity distribution in simulation and in the experiment, measured at different distances away from the light source.

To determine the similarity between the distribution of intensity in simulation and experiment, normalized cross correlation (NCC) was applied [33,34], which can be written as:

(3) $$NCC=\frac{\sum_{n}\left(I{\left(S_{n}\right)}_{d}-{\bar{I}}_{d}\right)\left(I{\left(S_{n}\right)}_{r}-{\bar{I}}_{r}\right)}{\sqrt{\sum_{n}{\left(I{\left(S_{n}\right)}_{d}-{\bar{I}}_{d}\right)}^{2}\sum_{n}{\left(I{\left(S_{n}\right)}_{r}-{\bar{I}}_{r}\right)}^{2}}}$$

where *I_d_* and *I_r_* are the simulated value and the measured value, respectively, ${\bar{I}}_{d}$ and ${\bar{I}}_{r}$ is the average value of *I_d_* and *I_r_*, and *S_n_* is the distance between the nth point and the optical axis. The calculated values of NCC of the blue curve and light blue curve in Figure 5 are both higher than 99.23%. At a certain distance away from the light source, namely 25 mm, 50 mm, 75 mm, 100 mm, 150 mm, 200 mm, 250 mm and 300 mm, the intensity distribution in simulation and experiment is also compared by NCC, respectively. As shown in Figure 6, NCC is higher than 98% in all cases, which means the correctness and effectiveness of simulation with the above method.

### 4.2. Calculation of the Freeform Surface Lens

Before designing the freeform surface lens, the essential initial parameter needs to firstly be determined, namely the thickness *d*, refractive index *n_L_*, focus length *f* and aperture D.

#### 4.2.1. Thickness d and Refractive Index n_L_

**Figure 7:**
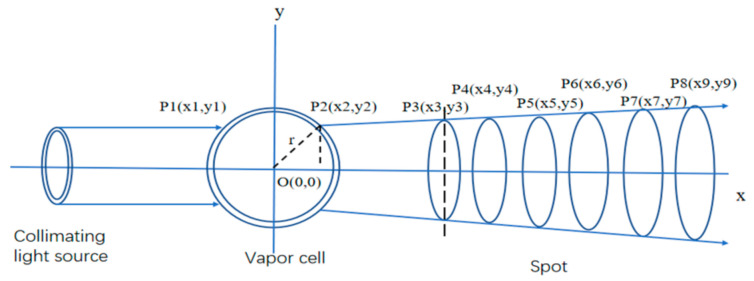
Solution to obtain the thickness and refractive index of the freeform surface lens.

**Figure 8:**
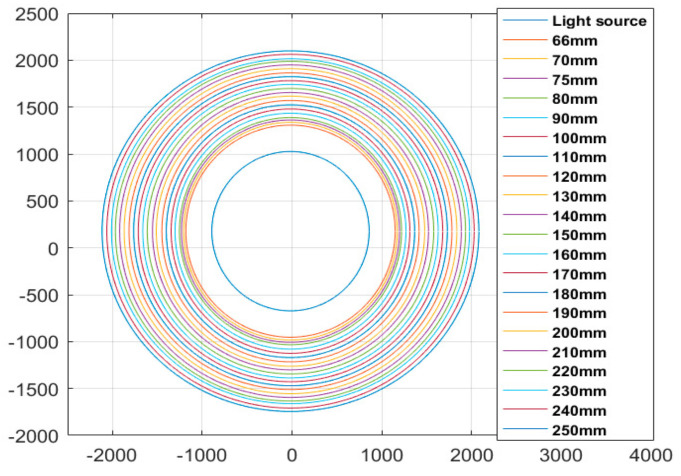
The spot measured at different distances away from the light source.

The solution to obtain the thickness and refractive index is shown in Figure 7. Firstly, the coordinate values of points (P3, P4…P8) were obtained by using a beam quality analyzer, as shown in Figure 8, and the relationship between the horizontal ordinate x and vertical coordinate y was then calculated by the least square method, namely:

(4) y=0.005087x+0.829

The R-square of the fitting was calculated as 0.9998. In the case with line P2P8 intersecting the spherical air cell, *x*2 and *y*2 can not only be satisfied by Equation (4), but also can obey the following equation:

(5) $$x^{2}+y^{2}=r^{2}$$

where *r* is the radius of the vapor cell and it is 7 mm. According to Equations (4) and (5), the coordinates of P2 are calculated to be 0.5550, 0.8318.

In the TracePro software, the collimating light source and vapor cell were established, and the thickness d and refractive index *n_L_* were simulated to be 0.165mm and 1.476, which ensured that the marginal ray of spot just appeared to be refracting at the point of P2.

#### 4.2.2. Focus Length f

According to the geometric optics, if the refractive index n in object space and n’ in image space are both equal to 1, the following relation can be obtained:

(6) $$f=f^{\prime }=\frac{1}{\left(n_{L}-1)(\frac{1}{r}-\frac{1}{r_{1}}\right)}$$

where *r*_1_ = *r* − *d*. *r*_1_ is then calculated to be 6.8 mm, and the focal length *f* is 609.2 mm.

#### 4.2.3. Aperture D

**Figure 9:**
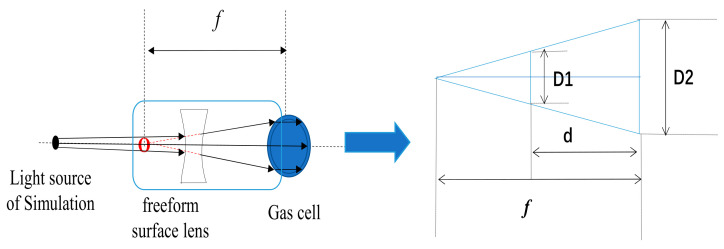
Calculation of the aperture of the freeform surface lens.

As shown in Figure 9, *D*1 is the aperture of the freeform surface lens, and *D*2 is the diameter of the vapor cell, and *d* is the distance between the freeform surface lens and the alkali metal vapor cell. In our work, *D*2, *d* and *f* were 14.0 mm, 550.0 mm and 609.2 mm, respectively. *D*1 can be calculated as follows:

(7) $$\frac{f-d}{f}=\frac{D1}{D2}$$

*D*1 was then calculated to be 1.36 mm.

### 4.3. Thickness Optimization Design of the Freeform Surface Lens

**Figure 10:**
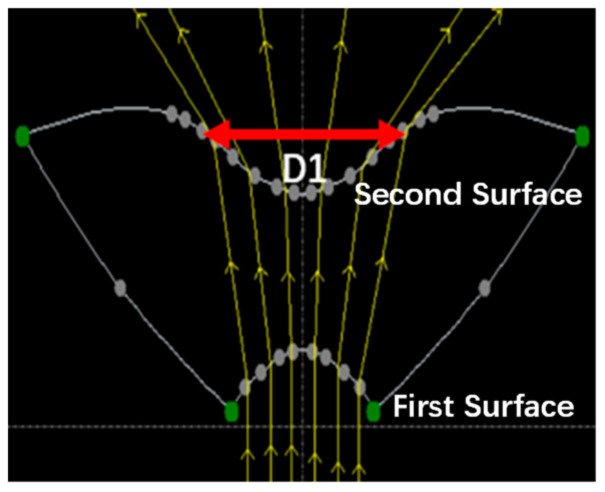
The established structure of the freeform lens in TracePro.

By using the interactive optimizer of the TracePro software, the optical system arranged as shown in Figure 1 was simulated, and the freeform surface lens was established with the determined parameters above. In Figure 10, the gray points on the two freeform surfaces were set as variables, and then the freeform surface lens was optimized with a global and local optimization process, until the desired intensity distribution was achieved [35].

**Figure 11:**
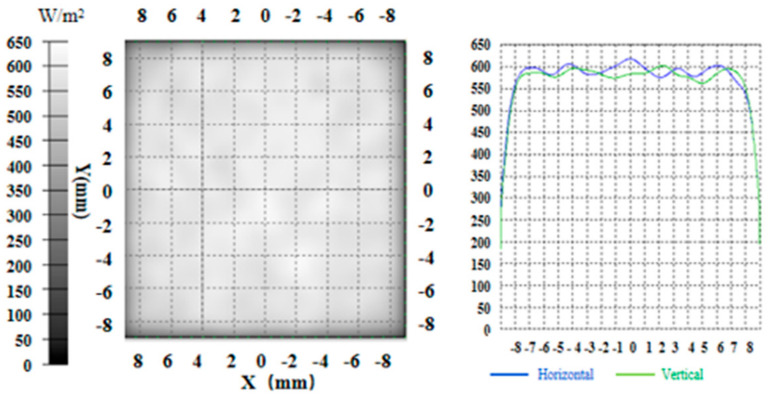
The optimized intensity distribution in the simulation.

Finally, the optimized intensity distribution is shown in Figure 11, and the uniformity was calculated to be 0.9534 according to Equation (2), and the uniform light spot with a radius of 7 mm was used to polarize the atoms.

**Figure 12:**
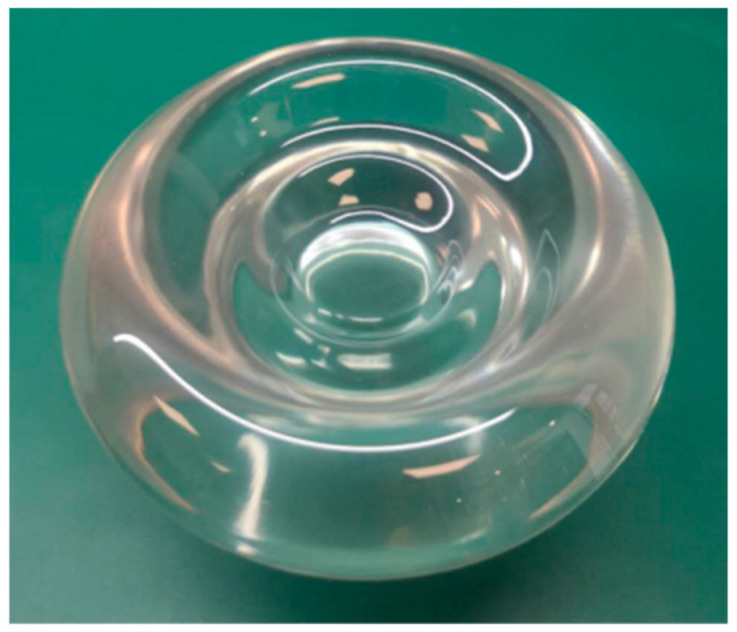
The actual designed freeform surface lens.

**Figure 13:**
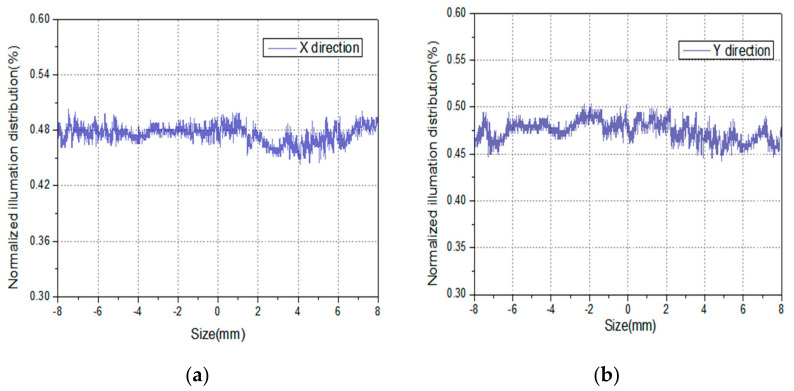
The illuminance distribution curves along X, Y directions in the experiment. (**a**) X direction, and (**b**) Y direction.

In Figure 12, the freeform surface lens was manufactured by the injection molding method by Suzhou Phelp Precision Model Co. Ltd. The freeform surface lens was made of polymethyl methacrylate (PMMA), and the machining accuracy was about 0.02 mm. The aperture and thickness were set to be 1.36 mm and 7.00 mm. Since the detection area of the beam quality analyzer was only 8.8 × 6.6 mm^2^, a displacement table of LX20/M (Thorlabs Company) was used to make the beam quality analyzer move in X and Y directions, respectively, to measure an area of 16 × 16 mm^2^. By applying the designed freeform surface lens, the intensity distribution along the X and Y directions was measured in the experiment, as shown in Figure 13. The uniformity along the two directions was calculated to be both higher than 90% according to Equation (2). Since the machining accuracy of the freeform surface lens was 0.01 mm and the designed lens aperture was only 1.36 mm, the error of 0.01 mm also caused the change of the lens surface shape and introduced the machining error, resulting in the uniformity in the actual experiment being slightly lower than that of the simulation.

## 5. Conclusions

We applied one scheme to achieve the square wave modulation and power stability control of the pumping laser, and the other one to obtain the uniform intensity distribution of the laser beam. Firstly, the feedback control of power based on the first-order diffraction light of the AOM and PID controller was achieved, and the power stability was controlled to be within 0.13%. Secondly, a substituted method of the light source was proposed, and the NCC of the distribution of the intensity in the simulation and measurement was calculated to be higher than 98% at different distances away from the light source. Finally, a freeform surface lens was optimized in the TracePro software and manufactured in actuality; the intensity uniformity of the light spot in the simulation and experiment were both higher than 90% on the target surface with a diameter of 14 mm.

## Data Availability

All data generated or analyzed during this study are included in this article.
